# The molecular mechanisms of inflammation and scarring in the kidneys of immunoglobulin A nephropathy: Gene involvement in the mechanisms of inflammation and scarring in kidney biopsy of IgAN patients

**DOI:** 10.1007/s00281-021-00891-8

**Published:** 2021-10-21

**Authors:** Francesco Paolo Schena, Michele Rossini, Daniela Isabel Abbrescia, Gianluigi Zaza

**Affiliations:** 1 Department of Emergency and Organ Transplant, University of Bari, Bari, Italy; 2 Schena Foundation, Policlinic, Bari, Italy; 3 Department of Nephrology, University of Verona, Verona, Italy

**Keywords:** Transcriptomics, Immunoglobulin A nephropathy, Kidney biopsy, Urine

## Abstract

Kidney biopsy is the cornerstone for the diagnosis of immunoglobulin A nephropathy (IgAN). The immunofluorescence technique evidences the IgA deposits in the glomeruli; the routine histology shows degree of active and chronic renal lesions. The spectrum of renal lesions is highly variable, ranging from minor or no detectable lesions to diffuse proliferative or crescentic lesions. Over the past three decades, renal transcriptomic studies have been performed on fresh or frozen renal tissue, and formalin-fixed paraffin-embedded kidney tissue specimens obtained from archival histological repositories. This paper aims to describe (1) the transcriptomic profiles of the kidney biopsy and (2) the potential urinary biomarkers that can be used to monitor the follow-up of IgAN patients. The use of quantitative Real-Time Polymerase Chain Reaction (qRT-PCR), microarrays and RNA-sequencing (RNA-seq) techniques on renal tissue and separated compartments of the nephron such as glomeruli and tubule-interstitium has clarified many aspects of the renal damage in IgAN. Recently, the introduction of the single-cell RNA-seq techniques has overcome the limitations of the previous methods, making that it is possible to study the whole renal tissue without the dissection of the nephron segments; it also allows better analysis of the cell-specific gene expression involved in cell differentiation. These gene products could represent effective candidates for urinary biomarkers for clinical decision making. Finally, some of these molecules may be the targets of old drugs, such as corticosteroids, renin–angiotensin–aldosterone blockers, and new drugs such as monoclonal antibodies. In the era of personalized medicine and precision therapy, high-throughput technologies may better characterize different renal patterns of IgAN and deliver targeted treatments to individual patients.

## Introduction

Immunoglobulin A nephropathy (IgAN) is characterized by abnormal production of galactose-deficient immunoglobulin A1 (Gd-IgA1), a non-self-antigen leading to anti-glycan antibodies. The result is high levels of circulating immune complexes (Gd-IgA1-IgG or IgA) in the blood. They localize at renal level and bind the mesangial cells through specific receptors with further deposition in the mesangium of glomeruli.

IgA1-immune complexes in the kidney could cause local production of cytokines, chemokines, and growth factors, responsible for glomerular lesions. In the first phase of the disease, the inflammatory process involves the mesangium, causing mesangial cell proliferation and abnormal production of the mesangial matrix. The inflammation affects the endothelial capillary cells, podocytes, and tubular cells. The final step is the fibrosis process, involving glomeruli (glomerular sclerosis) and tubule-interstitium (interstitial fibrosis), leading to a gradual decline of the renal function.

This paper aims to describe (1) the transcriptomic profiles of the kidney biopsy and (2) the potential urinary biomarkers that can be used to monitor the follow-up of IgAN patients.

## Renal transcriptomics

### Strategies for transcriptomic studies

Over the past three decades, renal transcriptomic studies have been performed on fresh or frozen whole renal tissue and formalin-fixed paraffin-embedded (FFPE) kidney tissue specimens obtained from archival histological repositories. Whole kidney tissue and separated renal compartments, such as isolated glomeruli or tubule-interstitium, have been examined.

In the early studies, manual microdissection of glomeruli was performed using a needle under a stereomicroscope and then under the laser capture microscope. First, manual microdissection of the nephron segments was performed but the manual skill, the time required for microdissection, and the loss of mRNA integrity during the microdissection limited this technique. Next, the use of laser capture microdissection that overcome these limitations increased the application of this technique.

The renal transcriptome shows all RNA transcripts, including mRNAs (ribosomal and transfer RNA) and regulatory non-coding RNAs. The researchers, first, used the qRT-PCR methods, then, microarray chips, and, recently, RNA-seq techniques which include bulk methods or single-cell techniques on isolated glomeruli and tubules or whole tissue.

In the era of personalized medicine and precision therapy, high-throughput technologies may better characterize different renal patterns of the disease and deliver targeted treatments to individual patients.

### Transcriptomics in fresh whole renal tissue

The first studies on gene expression profiles were conducted on the cortical section of kidneys obtained from patients with renal cell carcinoma who underwent nephrectomy and from kidney biopsies of IgAN patients (Table 1). Yano et al. [1] described, for the first time, the transcriptome profile of the kidney biopsy in IgAN patients. They used a high-density cDNA microarray with 18,326 paired unique human cDNA gene probes to study the different mRNA expression of genes. The computational analysis demonstrated various gene profiles in IgAN patients in different grades of renal pathology. Next, Waga et al. [2] used mRNA differential display to compare transcriptomic profiling of renal biopsies from IgAN patients and other glomerulonephrites to a normal part of kidney obtained after nephrectomy from patients with renal cell carcinoma. RNA samples from IgAN patients were pooled into four groups according to the degree of renal damage (from mild to severe). They used restriction endonucleolytic analysis of differentially expressed sequences (READS) and qRT-PCR. Statistical analysis of changes in gene expression identified 13 genes that were elevated in IgAN. Some of them varied with the clinical activity of the disease documented by the renal lesions. Next, using cluster analysis, the investigators identified three clusters of genes. The Rsp5gene, identified as EST 22402, an ubiquitin ligase for RNA polymerase II, and a 45 kD protein were found to be upregulated. Both overexpressed genes can change the functional activity of a wide range of proteins and contribute to the disease’s development.

**Table 1:** Transcriptomic profile in frozen whole renal tissue, FFPE renal tissue, and in isolated glomeruli and tubule-interstitium

Authors	Year	Methodology	Renal tissue	DEGs	Main findings
				Whole renal tissue	
Yano et al.	2002	HD cDNA microarray hybridization	OCT-Frozen	↑ MAPK1, CRC protein, PTDSS, HLA-E, PLEK P47, FAP, ANXA4, MUC18, ELN, DHFR, KRAS, RBR107, PFKM, PLD, GRM, ZNF33A	Different gene profiles related to different grades of renal damage
Waga et al.	2003	Restriction endonucleolytic	OCT-Frozen	↑ EST22972, MBP45K, GN01, EST22360, ubiquitin, gp330, prolyl2, E4TF1-47, CathD, Zinc29in4628, PDI, EST43719, OSF2	Megalin may be a potential drug receptor
Lepenies et al.	2010	qRT-PCR	Frozen	↑ PPARγ, IL-6, TGFβ-1, MCP-1	PPARγ increases with loss of the renal function
Lepenies et al.	2011	qRT-PCR	Frozen	↑ TLR4, MCP-1, TGFβ-1, IL-6	TLR4 may be a significant mediator of inflammation and fibrotic renal injury
Brabcova et al.	2011	qRT-PCR	Frozen	↑ CCL2, CCL5, TGFβ-1, HGF, BMP7	TGFβ-1 is associated with advanced vasculopathy and fibrogenesis
Rudnicki et al.	2012	qRT-PCR	OCT-Frozen	↑ Versican, TGFβ-1	Versican increases the renal disease progression
Cox et al.	2020	Microarrays	FFPE	↑ DEFA4, TNFAIP6, FAR2, LTB, CXCL6, ITGAX	TNFAIP6 (active renal tissue); CXCL6 (chronic renal tissue)
				Microdissected glomeruli and tubule-interstitium	
Liu et al.	2017	Microarray	G-TI	25 differentially expressed pathways from microarray data	Gene profile in mesangial cells
Ebefors et al.	2011	Microarray	G-TI	*Glomeruli* ↑ Biglycan, Decorin, Perlecan, NDST1, TGF-β1 *Tubules* ↓ Perlecan, VEGF	Increased expression of perlecan indicates better outcome
Tao et al.	2020	Microarray	G	↑ IFNG, pSTAT1, STAT, JAK1	JAK-STAT signaling pathway
Hodgin et al.	2014	Microarray	G	↑ AURKB, C1QA, C1QB, C2, CD163, CD44, CORO1C, FOXM1, GPR183, HPSE, KIF15, LAIR1, LAPTM5, LHFPL2, MAN2B1, MERTK, MS4A6A, PRC1, SLC43A3, TIMP1, VSIG4	Innate immune response, activation of the classical complement pathway, matrix degradation and turnover, macrophage polarization
Ju et al.	2009	Microarray	G-TI	NCF2, MPV17I, S100A6, BGN, COL6A1, ITGB5, SLC13A3, DKK3	Gene expression signature that predicts progressive renal fibrosis and CKD
Eikmans et al.	2003	qRT-PCR	G-TI	*Glomeruli* ↑ TGF-β, collagen I, collagen IV, fibronectin *Tubules* ↑ TGF-β	TGFβ may be a promising biomarker of renal outcome
Rudnicki et al.	2007	Microarray	TI	↓ TIMP1, DUSP5; ↓ BIK, TAX1BP1, TIA1 (apoptosis); ↑ BMP7, DEFB1, RAB4A (cell differentiation); ↑ THBS3, ADAM22 (cell adhesion and differentiation); ↑ IGF1, CDC34, ANXA13, RRAS, MARK3 (cell proliferation and cell cycle control); ↑ DEFB1, IFNGR1, C1QR1, SERP1, TPST1 (immune response); ↑ RAB4A, RAB43, SNX2, SEC6L1, SEC14C1, TOMM7 (intracellular transport); ↑ TCN1, KCND3, AQP2, AQP4 (membrane transport); ↑ PTPN1, ROS1, PRKD3, HSPC121, CSNK1D, COPS6, NMU, FRAT1 (signal transduction)	Proteinuria is associated to injury and repair mechanism
Reich et al.	2010	Microarray	TI	↑ COL1A1, EGR1, ELF3, IER3, HBEGF, MAFF, MCL1, SAMD4A, PAI-1, STEAP1, TYMS	Proteinuria signature at tubular level, apoptosis of tubular cells, cell-to-cell signaling, cell cycle, renal fibrosis
Ju et al.	2015	Microarray	TI	↑ NNMT, EGF, TMSB10, TIMP1, TUBA1A, ANXA1	Prediction of kidney function
Shved et al.	2017	Microarray	G-TI	↑ ABCG2, MXI1, NDRG1, RORA, VEGFA, BNIP3; ↓ BHLHE41, CXCR4, EDN1, HIF1A, ITGB2, MCL1, MET, PFKFB3	HIF-target genes correlated with renal function

ABCG2, ATP binding cassette subfamily G member 2; ADAM22, A Disintegrin And Metalloproteinase domain 22; ANXA1, Annexin A1; ANXA4, Annexin A4; ANXA13, Annexin A13; AQP2, Aquaporin 2; AQP4, Aquaporin 4; AURKB, Aurora Kinase B; BGN, Biglycan; BHLHE41, Basic Helix-Loop-Helix Family Member E41; BIK, BCL2-interacting killer (apoptosis inducing); BNIP3, BCL2/Adenovirus E1B 19 kDa Interacting Protein 3; BMP7, Bone Morphogenic Protein-7; C1QA, Complement C1q A Chain; C1QB, Complement C1q B Chain; C1QR1, Complement component 1, q subcomponent, and receptor 1; C2, Complement 2; CATHD, Cathepsin D; CCL2, C–C motif chemokine ligand 2; CCL5, C–C motif chemokine ligand 5; CD44, CD44 molecule; CD163, CD163 molecule; CDC34, Cell division cycle 34; CKD, Chronic Kidney Disease; COL1A1, Collagen Type I Alpha 1 chain; COL6A1, Collagen type VI, α1; COPS6, COP9 constitutive photomorphogenic homolog subunit 6; CORO1C, Coronin 1C; CRC protein, Colorectal cancer protein; CSNK1D, Casein kinase 1, delta; CXCL6, C-X-C motif chemokine ligand 6; CXCR4, C-X-C motif Chemokine Receptor 4; DEFA4, Defensin Alpha 4; DEFB1, Defensin, beta-1; DHFR, Dihydrofolate Reductase; DKK3, Dickkopf 3; DUSP5, Dual specificity phosphatase 5; E4TF1-47 or GABPB1, GA binding protein transcription factor subunit beta-1; EDN1, Endothelin 1; EGF, Epidermal Growth Factor; EGR2, Early growth response 1; ELF3, E74-like factor 3 (ets domain transcription factor, epithelial specific); ELN, Elastin; EST22360, Expressed sequence tag 22360; EST22972, Expressed sequence tag 22972; EST43719, Expressed sequence tag 43719; FAP, Fibroblast Activation Protein Alpha; FAR2, Fatty Acyl-CoA Reductase 2; FFPE, Formalin Fixed Paraffin Embedded; FOXM1, Forkhead Box M1; FRAT1, Frequently rearranged in advanced T-cell lymphomas; G, Glomeruli; GN01 or STARD10, StAR-Related Lipid Transfer Domain-Containing 10; GP330, Glycoprotein 330; GPR183, G Protein-Coupled Receptor 183; GRM, Glutamate Metabotropic Receptor; HBEGF, Heparin-binding EGF-like growth factor; HD, High Density; HGF, Hepatocyte Growth Factor; HIF1A, Hypoxia-Inducible Factor 1 Subunit Alpha; HLA-E, Major Histocompatibility Complex, Class I, E; HPSE, Heparanase; HSPC121 or BIND1, Butyrate-induced transcript 1; IER3, Immediate early response 3; IFNG, Interferon Gamma; IFNGR1, Interferon gamma receptor 1; IGF1, Insulin-like growth factor 1; IL-6, Interleukin-6; ITGAX, Integrin Subunit Alpha X; ITGB2, Integrin Subunit Beta 2; ITGB5, Integrin Subunit Beta 5; JAK1, Janus kinase 1; KIF15, Kinesin Family Member 15; KCND3, Potassium voltage-gated channel, Shal-related subfamily, member 3; KRAS, KRAS Proto-Oncogene, GTPase; LAIR1, Leukocyte-Associated Immunoglobulin-Like Receptor 1; LAPTM5, Lysosomal Protein Transmembrane 5; LHFPL2, Lipoma HMGIC Fusion Partner-Like 2 Protein; LTB, Lymphotoxin beta; MAFF, V-MAF Musculoaponeurotic fibrosarcoma oncogene homolog F; MAN2B1, Mannosidase Alpha Class 2B Member 1; MAPK1, Mitogen-activated protein kinase 1; MARK3, MAP/microtubule affinity-regulating kinase 3; MBP45K, 45 kD protein megalin-binding protein; MCL1, Myeloid cell leukemia sequence 1 (BCL2-related); MCP-1, Monocyte Chemoattractant Protein-1; MERTK, MER Proto-Oncogene, Tyrosine Kinase; MET, Mesenchymal Epithelial Transition, Proto-Oncogene, Receptor Tyrosine Kinase; MPV17l, MPV17 Mitochondrial Inner Membrane Protein Like; MS4A6A, Membrane Spanning 4-Domains A6A; MUC18, Cell Surface Glycoprotein MUC18; MXI1, MAX Interactor 1, Dimerization Protein; NCF2, Neutrophil Cytosolic Factor 2; NDRG1, N-Myc Downstream-Regulated 1; NDST1, N-deacetylase/N-sulfotransferase 1; NMU, Neuromedin U; NNMT, Nicotinamide N-Methylransferase; OCT, Optical Coherence Tomography; OSF2, Osteoblast-specific factor 2; PAI-1, Plasminogen Activator Inhibitor 1 (or Serpine1—Serpin Family E Member 1); PDI, Protein disulfide isomerase; PFKFB3, 6-Phosphofructo-2-Kinase/Fructose-2,6-Biphosphatase 3; PFKM, Phosphofructokinase, Muscle; PLD, Phospholipase D; PLEK P47, Pleckstrin P47; PPARγ, Peroxixome Proliferator-Activated Receptor gamma; PROLYL 2, Prolyl hydroxylase 2; pSTAT1, phospho-Signal Transducer and Activator of Transcription 1; PRC1, Protein Regulator of Cytokinesis 1; PRKD3, Protein kinase D3; PTDSS, Phosphatidylserine Synthase; PTPN1, Protein tyrosine phosphatase, non-receptor type 1; qRT-PCR, quantitative Real-Time Polimerase Chain Reaction; RAB4A, RAB4A, member RAS oncogene family; RBR107, Retinoblastoma-related protein; RORA, RAR-Related Orphan Receptor A; ROS1, V-ros UR2 sarcoma virus oncogene homolog 1; RRAS, Related RAS viral (r-ras) oncogene homolog; S100A6, S100 calcium-binding protein A6; SAMD4A, Sterile alpha motif domain-containing 4A; SEC6L1, SEC6-like 1; SEC14C1, SEC14-like 1; SERP1, Stress-associated endoplasmic reticulum protein 1; SLC13A3, Solute Carrier Family 13 member 3; SLC43A3, Solute Carrier Family 43 Member 3; SNX2, Sorting nexin 2; STAT, Signal Transducer and Activator of Transcription; STEAP1, Six transmembrane epithelial antigen of the prostate 1; TAX1BP1, Tax1 (human T-cell leukemia virus type I) binding protein 1; TCN1, Transcobalamin I (vitamin B12 binding protein, R binder family); TGF-β1, Transforming Growth Factor Beta-1; THBS3, Thrombospondin-3; TI, Tubule-Interstitium; TIA1, TIA1 cytotoxic granule-associated RNA-binding protein; TIMP1, Tissue Inhibitor of Metalloproteinase 1; TLR4, Toll Like Receptor 4; TMSB10, Thymosin β 10; TNFAIP6, TNF Alpha-Induced Protein 6; TOMM7, Translocase of outer mitochondrial membrane 7 homolog; TPST1, Tyrosylprotein sulfotransferase 1; TUBA1A, Tubulin α 1A; TYMS, Thymidylate synthetase; VEGFA, Vascular Endothelial Growth Factor A; VSIG4, V-Set and Immunoglobulin Domain-Containing 4; ZINC29IN4628, Human small zinc finger-like protein; ZNF33A, Zinc Finger Protein 33A

In subsequent years, researchers studied the gene expression profile of inflammatory molecules in the kidney biopsies of IgAN patients to identify biomarkers involved in the progression of renal damage (Table 1). They used qRT-PCR that involves three steps: (1) reverse transcription of mRNA into cDNA, (2) amplification of specific cDNA sequences using PCR, and (3) quantification of amplified products.

Perixome proliferator-activated receptor gamma (PPARγ) is considered a renoprotective factor in type 2 diabetic nephropathy. In fact, its agonist reduces proteinuria. Using qRT-PCR, Lepenies et al. [3] studied the mRNA expression of this receptor in kidney biopsies of patients with chronic kidney disease, 13 of which were from patients with IgAN. The mRNA expression of other cytokines [Monocyte Chemoattractant Protein-1 (MCP-1), Transforming Growth Factor-β1 (TGF-β1), Interleukin-6 (IL-6)] was also investigated. A significantly increased expression of PPARγ was found in patients with impaired renal function. The high expression of this receptor correlated positively with MCP-1 and negatively with TGF-β1 expression. These results suggested that the use of PPARγ agonists may have a protective effect in patients with impaired renal function, and potentially IgAN patients may benefit from the administration of thiazolidinedione molecules such as rosiglitazone and pioglitazone. Later, the same investigators [4] studied in the same cohort of patients, including 14 IgAN, the mRNA expression of Toll-like receptor 4 (TLR4) which would be associated with other inflammatory mediators, increased proteinuria, impaired renal function, and progression of the kidney damage. They extracted total RNA from the renal tissue after the kidney biopsy and measured by qRT-PCR the expression of TLR4 and other inflammatory factors such as MCP-1, IL-6, and TGF-β1. Patients with IgAN had a substantially high expression of TLR4, which correlated with MCP-1, IL-6, and TGF-β1 mRNA expression. This expression increased in the presence of high proteinuria. Thus, TLR4 appeared to be a significant mediator of inflammatory and fibrotic renal injury.

Using the same technical approach, Brabcova et al. [5] measured the mRNA expression of TGF-β1 and other cytokines such as (C–C motif) ligand 2 (CCL2), (C–C motif) ligand 5 (CCL5), hepatocyte growth factor (HGF), and bone morphogenic protein-7 (BMP7) in kidney biopsies of 51 IgAN patients. Results showed that the mRNA expression of these inflammatory molecules correlated significantly with the interstitial infiltrate of T-cells (CD3^+^), cytotoxic T-cells (CD8^+^), B cells (CD20^+^), and macrophages (CD68^+^). Moreover, the overexpression of the inflammatory molecules was associated with increased proteinuria. Next, the investigators studied the behavior expression of these molecules in a longitudinal study, including some patients in the cohort. They found higher renal expression of TGF-β1 and CD68 in patients called progressors. Finally, advanced chronic vasculopathy and higher TGF-β1 expression were associated with two-year disease progression. In conclusion, the interstitial infiltrate of lympho-monocytes in the kidneys of IgAN patients correlated with mRNA overexpression of the inflammatory molecules that participate in the vasculopathy and fibrosis of the renal structures.

In a cohort of 74 patients, of whom 19 were affected by biopsy-proven IgAN, Rudnicki et al. [6] studied the expression of Versican isoforms (V0 and V1), extracellular matrix proteins of the connective tissue, using the qRT-PCR on RNA extracted from archival frozen kidney tissue. They found an increased expression of the two isoforms in kidney biopsies of patients with progressive decline of the renal function. This biomarker correlated positively with serum creatinine and degree of histological damage; thus, it may be considered an expression of unfavorable clinical outcome.

### Transcriptomics in FFPE-whole renal tissue

FFPE renal tissue samples can represent a valuable alternative to the fresh or frozen renal tissue in the study of molecular biology. Donczo and Guttman [7] defined this archival tissue sample as the Holy Grail for molecular diagnostics. Formalin fixation, temperature, and time of conservation are critical points in maintaining the integrity of nucleic acids (DNA and RNA). Still, only small fragments of mRNA are required to study the gene expression profile of genes. However, Ribeiro-Silva et al. [8] demonstrated that degraded RNA obtained from FFPE tissue samples was successfully used in gene expression studies. The integrity of RNA is essential for gene expression studies; therefore, RNA integrity (RIN) is evaluated using agarose gel electrophoresis. Today, the automated Agilent 2100 bioanalyzer is currently used to estimate the RNA integrity (RIN) [9].

Cox et al. [10] studied the gene expression of whole tissue in FFPE kidney biopsies from a cohort of 52 IgAN patients using the whole-genome cDNA-mediated annealing, selection, extension, and ligation (DASL) HT assay. Bioinformatic analysis identified specific transcripts in IgAN patients with active (endocapillary and extracapillary lesions) and chronic (tubule-intestitial damage) renal lesions (Table 1). The network analysis of active lesions identified 35 differentially expressed genes (DEGs) of which 18 were representative of the glucocorticoid signaling pathway. These data indicate a potential influence of corticosteroids in patients with active renal lesions. The analysis highlighted two genes Defensin Alpha 4 (DEFA4) and TNF Alpha-Induced Protein 6 (TNFAIP6) in active renal lesions. In kidney biopsies with active renal lesions, the protein expression of these two genes was upregulated in glomerular cells. The top-ranked network for chronic renal lesions was represented by 33 genes. Many extracellular chemokines such as Lymphotoxin beta (LTB) and Chemokine (C-X-C motif) ligand 6 (CXCL6) and another gene Integrin Subunit Alpha X (ITGAX) involved in cell adhesion and infiltration were found upregulated. These genes were confirmed by qRT-PCR and immunohistochemistry in chronic renal lesions at the glomerular and tubular levels. In conclusion, specific markers for active and chronic renal lesions were evidenced in kidney biopsies of IgAN patients.

### Transcriptomics in microdissected glomeruli and tubule-interstitium

The structural compartmentalization of the kidney has stimulated investigators to separate glomeruli from tubules and interstitium and study the gene expression of the distinct nephron segments for a better comprehensive molecular characterization related to their functions and participation in the kidney damage. A multicenter study demonstrated that the separation of 10% of a renal biopsy core and accurate mRNA protection for storage did not significantly interfere with routine diagnostics [11]. On behalf of the project on chronic renal disease granted by the 5th European Framework, the first European Renal cDNA Consortium was constituted in collaboration with the Kroener-Fresenius biopsy bank (ERCB) [12]. From the diagnostic fresh renal biopsy, a segment of the specimen not used for diagnostic evaluation was processed for microdissection and gene expression studies, after informed consent of the patients and approval of the local ethics committees.

#### Microarray technique and qRT-PCR

The microarray technique is based on the use of chips that contain a pre-specified set of nucleic acid probes bound to glass slides that recognize specific RNA sequences. The fluorescent hybridization process is evaluated by quantifying the signal intensity. The limit of this technique is due to a fixed number of nucleic acid probes that are not regularly updated.

The mesangium of glomeruli is the final site where there is the deposition of circulating immune complexes with sequential mesangial cell proliferation and mesangial matrix expansion (Table 1). Therefore, it is vital to know the role of mesangial cells in the development of the IgAN. Liu et al. [13] investigated on the gene expression of mesangium in microdissected glomeruli from kidney biopsies of IgAN patients. They found a distinct transcriptome pattern formed by 736 DEGs leading to 113 significant pathways. Most of them were related to inflammation, cytokines, and grow factors involved in an extracellular matrix organization and cell-to-cell adherence. Next, the investigators studied the gene profile in mesangial cells and podocytes and found a pattern of genes specific to the mesangial cells of IgAN patients. No difference was observed in the podocytes. The use of a mesangial cell-positive standard gene Z score found a correlation with serum creatinine, eGFR, and segmental glomerular sclerosis at the time of the kidney biopsy. These findings demonstrated the role of mesangial cells in the disease. In vitro proteomic study confirmed data in which isolated mesangial cells, after stimulation with Gd-IgA1, showed the same inflammatory pathways, including complement activation, in vitro and in vivo.

Ebefors et al. [14] studied the gene and protein expression of proteoglycans in microdissected glomeruli and tubule-interstitium from kidney biopsy of IgAN patients (Table 1). Proteoglycans are components of the mesangial matrix. They contribute to the charge selectivity of the glomerular basement membrane barrier. The investigators found at the glomerular level the overexpression of Perlecan (PRCAN), Biglycan (BGN), and Decorin (DCN), while Proteoglycan syndecan-1 (SDC1) was downregulated. At the tubular level, the expression of PRCAN was reduced. Proteinuria was inversely correlated with PRCAN expression in glomeruli while it correlated a tubular level with biglycan and decorin. Moreover, the renal function inversely correlated with the glomerular expression of PRCAN and nephrin (NPHS1). The overexpression of PRCAN and DCN was confirmed by immunohistochemistry in sclerotic glomeruli and tubule-interstitium. The glycan proteins correlated with global and segmental glomerular sclerosis and with tubule-interstitial compartment. Finally, patients with extracapillary lesions had a higher expression of the N-deacetylase/N-sulfotransferase 1 (NDST1) gene, which plays an essential role for heparan sulfate production. In conclusion, increased expression of PRCAN correlated with a better outcome of the patient in the presence of low levels of proteinuria.

Tao et al. [15] studied the role of the Janus kinase (JAK)-signal transducer and activator of transcription (STAT) pathway in the kidney damage of IgAN patients (Table 1). First, they observed using immunohistochemistry increased staining of STAT1, STAT3, phosphor (p)-STAT1, and pSTAT3. Therefore, they studied the transcriptome pattern of the JAK-STAT pathway in microdissected glomeruli and tubule-interstitium of kidney biopsies of IgAN patients. This pathway responds to extracellular signals, thus, producing cytokines and growth factors. An increased expression of the JAK-STAT signaling pathway was found in glomeruli, less in the tubule-interstitium. The researchers evaluated this activation by measuring the STAT1 activation score, composed of 17 genes. The STAT1 hyperactivation observed in IgAN patients was confirmed in another independent group of IgAN patients. However, the STAT1 activation was found in other primary and secondary glomerulonephritis, meaning that this process is generalizable to all glomerular diseases. The overexpression of STAT1 in the glomeruli correlated with high levels of serum creatinine, and elevated levels of proteinuria in the tubule-interstitium. A lower trend was observed in patients in complete remission even though it was not statistically significant. These results indicate that abnormal activation of the JAK-STAT pathway plays a vital role in the development of glomerular disease and its progression. Its inhibition may cause a reduction of proteinuria, as shown in diabetic nephropathy [16].

The active renal lesions of the IgAN are represented by endocapillary and extracapillary proliferation. Hodgin et al. [17] studied the molecular phenotype of the endocapillary proliferation in microdissected glomeruli from kidney biopsies of IgAN patients with (E1) and without (E0) endocapillary proliferation, obtained by the ERCB and the Toronto Glomerulonephritis Registry biobank (Table 1). They used transcriptome analysis and in silico drug screening. After the first filtering step, they managed a large number of DEGs, thus, obtaining the most representatives. The authors described the transcriptome pattern of the endocapillary lesions, characterized by 424 DEGs, of which 22 constituted a subset correlated with kidney function at the time of the kidney biopsy. Eight canonical pathways were expressed in biopsies with endocapillary lesions, mainly innate immune response and T-cell-signaling pathways. In addition, transcripts encoding the cell cycle and cell division were overexpressed. The transcripts encoding proteins involved in the innate immune response were associated with activation of the complement classical pathway, matrix degradation, and turnover. In addition, CD163, a marker of macrophages was upregulated and negatively correlated with renal function. Next, the investigators performed transcription factor analysis and found one quarter of the DEGs contained Nuclear Factor k-B (NFkB) consensus binding units that corticosteroids could inhibit. Tumor Protein P53 (TP53) and NFKB1 were the two top transcription factors having a binding site in the promoter of E1-upregulated genes. Finally, the authors used the in silico connectivity map approach and demonstrated methylprednisolone and cortisone downregulated 107 and 85 DEGs, respectively. They also identified other bioactive compounds such as cyclosporine, methotrexate, and hydroquinone. Moreover, the use of the Drug Pair Seeker tool showed that resveratrol, in combination with corticosteroids, was able to reverse the DEGs of the E1 lesions.

Ju et al. [18] studied the gene transcriptome of the tubule-interstitium compartment in 164 kidney biopsies, obtained by the ERCB, of which 24 exhibited IgAN (Table 1). The transcriptome correlated with the eGFR at the time of kidney biopsy. It was formed by 72 genes implicated in the Chronic kidney disease (CKD) progression. Next, it was validated by qRT-PCR in a second cohort of 55 kidney biopsies (14 IgAN). A panel of six genes [Nicotinamide N-methyltransferase (NNMT), Epidermal growth factor (EGF), Thymosin β 10 (TMSB10), Tissue Inhibitor of Metalloproteinases (TIMP1), Tubulin α1a (TUBA1A), and Annexin A1 (ANXA1)] had the best predictive performance. This panel was validated in another cohort of 42 patients with CKD; three genes (EGFR, NNMT, and TSMB10) formed the final panel showing the best eGFR prediction. Finally, the EGF gene was selected based on the best correlation with eGFR, protein renal expression, and biological mechanisms in the progression of renal damage. This growth factor plays an important role in the regeneration and repair processes. Therefore, the investigators measured the urinary levels of EGF in a large cohort of patients, and they found that this urinary biomarker inversely correlated with Interstitial fibrosis (IF)/Tubular atrophy (TA) score. Interestingly, both intrarenal EGFR expression and urinary levels correlated with the slope of eGFR. Therefore, the measurement of urinary levels of EGF improved the ability to predict the renal outcome. These findings confirmed our previous published data [19] in which we demonstrated that the urinary EGF/MCP-1 ratio was a predictor of progression of renal damage in IgAN patients.

The progression of renal damage in IgAN has been studied by many investigators. Eikmans et al. [20] quantized the mRNA levels of TGF-β, Collagen I (COL1A1) and IV (COL4A1), and Fibronectin (FN1) by qRT-PCR in separated compartments (glomeruli and tubule-interstitium) of kidney biopsies carried out in 52 patients with various chronic kidney diseases, of which 10 were IgAN, and 16 were controls (cadaveric kidney donors). They found that glomerular sclerosis and interstitial fibrosis were associated with higher expression of TGF-β, FN1, and COL4A1 at the glomerular level. There was also a significant increase of TGF-β in the tubule-interstitium compartment. The mRNA expression of these molecules correlated with lower eGFR and higher proteinuria. Moreover, they correlated with the progression of renal damage (Table 1). The authors concluded that the measurement of mRNA TGF-β levels in the renal tissue might be a promising predictor of the outcome because this growth factor moves to the repair process after the tissue injury.

Proteinuria causes the progression of renal damage. Rudnicki et al. [21] studied the mRNA expression of genes induced by proteinuria, using microarrays, in renal proximal tubular epithelial cells (Table 1). The genes were isolated using the Laser Capture Microdissection System and CapSure LCM caps in frozen kidney biopsies of 19 proteinuric patients, of whom eight were affected by IgAN. They found a decreased expression of some genes involved in apoptosis and high expression of other genes involved in cell adhesion and differentiation. Moreover, there was an increased expression of Insulin-like growth factor 1 (IGF1), genes involved in cell proliferation, cell cycle control, immune response, and genes involved in transport, metabolism, and matrix turnover. Next, the investigators selected five genes [Defensin beta-1 (DEFB1), Tissue inhibitor of metalloproteinase 1 (TIMP1), Dual specificity phosphatase 5 (DUSP5), BMP7 and Ras-related protein Rab-4A (RAB4A)] from the five different functional groups for the validation process. Two proteins, Thrombospondin-3 (TSP-3, cell adhesion) and BMP7 (cell differentiation), were chosen for the immunohistochemical localization in the kidney. Interestingly, a strong signal of BMP7 protein was found at the luminal side of epithelial cells. This study dissected the complex patho-physiological responses that developed in proteinuric IgAN and other nephropathies.

The harmful role of proteinuria was also studied by Reich et al. [22] who analyzed the gene expression of tubule-interstitium dissected from kidney biopsy of IgAN patients and the gene expression of primary human renal tubular cells after exposure to a medium containing 1% of bovine serum albumin for 6 h. The microarray study, conducted on primary human renal tubular cells, evidenced 231 DEGs, defined albumin-regulated genes, which were involved in different biological processes such as pro-inflammatory cell-signaling cytokines, cell cycling, apoptosis, connective tissue development, and fibrosis. Next, they extracted 49 DEGs, defined as the gene set from the microarray data of the renal tissue of IgAN patients using hierarchical cluster analysis. Finally, they studied the relationship between the expression levels of the 231 albumin-regulated genes in the tubule-interstitium compartment compared with the levels of proteinuria. Thus, the bioinformatic analysis selected 11 DEGs found in the tubule-interstitium of IgAN and other forms of primary glomerulonephritis. This set of genes was defined proteinuria signature. Finally, a transcriptional network was constructed using a natural language processing tool. Among the 11 DEGs, early growth response protein 1 (EGR1) was the central node linking the other 10 DEGs; many of them contained a proximal EGR1 promoter region consistent with a putative common transcriptional regulation. Principally, the overexpression of Plasminogen activator inhibitor-1 (PAI-1) and EGR1 was implicated in renal fibrosis at tubule-interstitial level (Table 1). In conclusion, a proteinuria signature of 11 genes was found in the tubule-interstitium of IgAN and other types of primary glomerulonephritis.

Many investigators studied the progression of the renal damage. Ju et al. [23] conducted a transcriptomic study in kidneys of TGF-β1-transgenic mice and developed a gene and protein signature (Table 1). They identified 43 genes that were validated in kidney tissue of older TGF-β1-transgenic mice and, then, in kidney biopsies of the same mice with progressive renal fibrosis. Finally, the investigators conducted a third validation study using 19 of 43 genes in cDNA samples of the ERCB, obtained from the renal tissue of 47 patients with chronic kidney disease, of whom 21 were affected by IgAN. They developed a panel of eight gene markers [Neutrophil cytosolic factor 2 (NCF2), Mitochondrial Inner Membrane Protein MPV17 (MPV17), S100 calcium-binding protein A6 (S100a6), Biglycan (BGN), Collagen type VI, α1 (COL6A1), Integrin β5 (ITGB5), Solute carrier family 13 member 3 (SLC13A3), and Dickkopf 3 (DKK3)] for the diagnosis of CKD progression that was confirmed by immunohistochemistry. In this manner, they distinguished patients with progressive and non-progressive kidney disease. However, these data were not validated in longitudinal studies.

Shved et al. [24] used microdissected glomeruli and tubule-interstitium to study hypoxia due to an imbalance between blood perfusion and oxygen demand in chronic kidney damage. The morphological expression of this dysregulated process is renal fibrosis. The cellular response to hypoxia is mediated by Hypoxia-inducible factors (HIF) present in glomerular and tubular cells. Transcriptome analysis carried out in IgAN patients and other glomerulonephrites with different CKD stages evidenced 24 interstitium HIF-target genes and 18 glomerular HIF-target genes, correlated with eGFR. The selected HIF-targets were confirmed using immunohistochemistry. An increased nuclear and cytoplasmic expression of HIF1a was observed more in tubules than in glomeruli in patients with reduced renal function. These data were confirmed by a microarray study carried out on in vitro tubular cells and podocytes when they were separately subjected to hypoxia (Table 1). In conclusion, an increased dysregulation of hypoxia-associated transcripts was observed in chronic kidney damage.

#### RNA-sequencing technique in isolated glomeruli

The RNA-seq method allows the sequence of all RNA including the splice isoforms present in the sample. This technique enables the transcriptomic study of the whole genome and detects high and low levels of gene expression with great precision. The RNA-seq techniques notably improves the study of the entire transcriptome, providing a better knowledge of all transcripts and splicing variants expressed by a cell type or specific tissue. Few articles have been published on kidney biopsies in IgAN patients, likely due to the high cost of this technique (Table 2).

**Table 2:** Transcriptomic patterns obtained by RNA-sequencing (RNA-seq) technique in isolated glomeruli and by Single Cell RNA-seq in whole renal tissue

Authors	Year	Methodology	DEGs	Main findings
			Isolated glomeruli	
Jiang et al.	2016	Illumina HiSeq 2000/2500	↑ JUN, FOS, PLAU (aging and inflammation); ↑ LL37 (senescent cells)	Renal infiltration of aging neutrophils causes IgA-immune complex deposition, renal injury, activation of RAAS, complement, and coagulation cascade
Park et al.	2020	Illumina HiSeq 2500	↑ CCL3, CCL4, CXCL16 (Inflammation); ↑ BTK, SYK, MAPK1 (B cell and Fcγ-R pathways); ↑ TNF, TGFBR1, SMAD3, SMAD5, COL1A1 (fibrosis); ↑ GRK2, HIF1A, PI3KCA (PI3K-Akt pathways); ↑ C4A, C4B (complement); ↑ B4GALT1 (mesangial receptor for IgA); ↓ DUSP1, MALAT1, CARMN, FOS ↓ FOSB, JUN (FOS-JUN pathway); ↓AGTR1, RGS2, RGS16, RGS11 (regulators of the inflammation)	SYK is increased in mesangial cells by signals initiated by IgA1. Thus, this molecule may be considered a potential target for specific drugs
			Whole renal tissue	
Zheng et al.	2020	STRT scRNA-seq	*Renal cortex—Resident cells* ↑ ATN1, PDGFRB, COL1A2; LUM (mesangial cells); ↑ PCOLCE2, NPHS2, WT1 (podocytes); ↑ AQP2, AQP3 (tubular cells); ↑ AQP6 (intercalated cells); ↑ SLC5A12, CUBN (proximal tubules); ↑ SLC12A1 (loop cells of Henle); ↑ JCHAIN (mesangial cells) *Renal cortex—Non-resident cells* ↑ CD3D, CD3E (T-cells); ↑ CD14, CD68, ITGAM (macrophages) *Inflammation process* ↑ THY1 (mesangial proliferation); ↑ Genes encoding collagen 5, glycoproteins, integrins; ↑ FN1 (ECM component that aids IgAN deposition); ↑ WFDC2 (protease inhibitor); ↑ SPP1, KNG1 (inflammatory mediators); ↑ PLGRKT, CCL2 (cytokines); ↑ AOC3 (leukocyte trafficking) *Chronic inflammation* ↑ PLGRKT2; ↑ CCL2, CX3CR1 (Macrophage activation); ↑ IFI44, IFI44L, IFI6, ISG15 (interferon response); ↑ FCGR3A, GZMB, KLRD, FGFBP2, GZMH (CD8^+^ T-cell cytotoxicity) *Intercalated cells and fibrosis* ↑ S100A4, ZEB1, ZEB2	ANGPT1/TEK interaction is the most prominent mesangial-endothelial cell–cell interaction
Tang et al.	2021	Illumina HiSeq X10	*Glomerular cells* ↑MALAT1, GADD45B, SOX4, EDIL3, FOS (mesangial cells); ↑ SOX4, MT-RNR1, PECAM1, UTRN, MT-ATP6P1, MT- ND4L (endothelial cells); ↑ PRSS23, NGF, HES1, (podocytes); ↑ SELP, PECAM1 (cell adhesion); ↑ SOX4, ACKR1, RNASE1 (endothelial cells) *Tubular cells* ↑ TNF, IL-17, NOD (epithelial cells); ↑ p38MAPK (distal tubule); ↑ ITGB6, ITGB8, YWHAH, SPP1, JUN, FOS (Henle cells); ↑ NFKBIA, TXNIP, CXCL3, CXCL2 (principal and intercalated cells) *Non-resident cells* ↓ GPX3, FAM49B, FCGBP (renal macrophages); *Cell–cell crosstalk* ↑ CXCL1, CCL2 (mesangial cells); ↑ ACKR1 (endothelial junctions); ↑ JAGGED1, NOTCH4 (endothelial cells); ↑ FGF2, PDGFD (proliferation and matrix production in mesangial cells) *Overt proteinuria* ↑ SPARC, ROCK2 (mesangial cells); ↑ TXNIP, SPARCL1, CD74 (endothelial cells)	The molecular inflammatory process moves from the mesangial cells to other cells combined with non-resident circulating cells. Proteinuria induces overexpression of genes at glomerular and tubular level leading to renal fibrosis
Chen et al.	2021	10X Genomix scRNA-seq	↑ MMP7, MYC (wnt-β catenin target genes activated in proximal tubular cells); ↑ FXYD5, CD74, B2M (podocytes); ↑ CLIC1, RPS26, LTB (mesangial cells)	Jun B gene may be a novel prognostic biomarker in IgAN

ACKR1, Atypical Chemokine Receptor 1; AGTR1, Angiotensin II Receptor Type 1; ANGPT1, Angiopoietin 1; AOC3, Amine Oxidase Copper-Containing 3; ATN1, Atrophin 1; AQP2, Aquaporin 2; AQP3, Aquaporin 3; AQP6, Aquaporin 6; B2M, Beta-2-Microglobulin; B4GALT1, Beta-1,4-Galactosyltransferase 1; BTK, Bruton Tyrosine Kinase; C4A, Complement C4A; C4B, Complement C4B; CARMN, Cardiac Mesoderm Enhancer-Associated Non-Coding RNA; CCL2, C–C Motif Chemokine Ligand 2; CCL3, C–C motif chemokine ligand 3; CCL4, C–C motif chemokine ligand 4; CD3D, T-Cell Surface Glycoprotein CD3 Delta Chain; CD3E, T-Cell Surface Glycoprotein CD3 Epsilon Chain; CD14, Monocyte Differentiation Antigen CD14; CD68, Macrophage Antigen CD68; CD74, CD74 Molecule; CLIC1, Chloride Intracellular Channel 1; COL1A1, Collagen Type I Alpha 1 Chain; COL1A2, Collagen Type I Alpha 2 Chain; CUBN, Cubilin; CX3CR1, C-X3-C Motif Chemokine Receptor 1; CXCL1, C-X-C Motif Chemokine Ligand 1; CXCL2, C-X-C Motif Chemokine Ligand 2; CXCL3, C-X-C Motif Chemokine Ligand 3; CXCL16, C-X-C motif chemokine ligand 16; DUSP1, Dual Specificity Phosphatase 1; ECM, Extra-cellular matrix; EDIL3, EGF-Like Repeats and Discoidin Domains 3; FAM49B, Family with Sequence Similarity 49, Member B; FCGBP, Fc Fragment Of IgG-Binding Protein; FCGR3A, Fc Fragment of IgG Receptor IIIa; FGF2, Fibroblast Growth Factor 2; FGFBP2, Fibroblast Growth Factor-Binding Protein 2; FN1, Fibronectin 1; FOS, Fos Proto-Oncogene, AP-1 Transcription Factor Subunit; FOSB, FosB Proto-Oncogene, AP-1 Transcription Factor Subunit; FXYD5, FXYD Domain-Containing Ion Transport Regulator 5; GADD45B, Growth Arrest and DNA Damage-Inducible Beta; GPX3, Glutathione Peroxidase 3; GRK2, G Protein-Coupled Receptor Kinase 2; GZMB, Granzyme B; GZMH, Granzyme H; HES1, Hes Family BHLH Transcription Factor 1; HIF1A, Hypoxia-Inducible Factor 1 Subunit Alpha; IFI6, Interferon Alpha-Inducible Protein 6; IFI44, Interferon-Induced Protein 44; IFI44L, Interferon-Induced Protein 44 Like; IgAN, Immunoglobulin A nephropathy; IL-17, Interleukin-17; ISG15, ISG15 Ubiquitin-Like Modifier; ITGAM, Integrin Subunit Alpha M; ITGB6, Integrin Subunit Beta 6; ITGB8, Integrin Subunit Beta 8; JAGGED1, Jagged Canonical Notch Ligand 1; JCHAIN, Joining Chain of Multimeric IgA and IgM; JUN, Jun Proto-Oncogene, AP-1 Transcription Factor Subunit; KLRD, Killer Cell Lectin-Like Receptor D; KNG1, Kininogen 1; LL37 or CAMP, Cathelicidin Antimicrobial Peptide; LTB, Lymphotoxin Beta; LUM, Lumican; MALAT1, Metastasis-Associated Lung Adenocarcinoma Transcript 1; MAPK1, Mitogen-Activated Protein Kinase 1; MMP7, Matrix Metallopeptidase 7; MT-ATP6P1, MT-ATP6 Pseudogene 1; MT-ND4L, Mitochondrially Encoded NADH:Ubiquinone Oxidoreductase Core Subunit 4L; MT-RNR1, Mitochondrially Encoded 12S RRNA; MYC, MYC Proto-Oncogene, BHLH Transcription Factor; NFKBIA, NFKB Inhibitor Alpha; NGF, Nerve Growth Factor; NOD, Nucleotide-Binding Oligomerization Domain Containing; NOTCH4, Notch Receptor 4; NPHS2, NPHS2 Stomatin Family Member, Podocin; p38MAPK, p38 Mitogen-Activated Protein Kinase; PCOLCE2, Procollagen C-Endopeptidase Enhancer 2; PDGFRB, Platelet-Derived Growth Factor Receptor Beta; PDGFD, Platelet-Derived Growth Factor D; PECAM1, Platelet and Endothelial Cell Adhesion Molecule 1; PI3KCA, Phosphatidylinositol-4,5-Bisphosphate 3-Kinase Catalytic Subunit Alpha; PLAU, Plasminogen Activator, Urokinase; PLGRKT, Plasminogen Receptor with A C-Terminal Lysine; PLGRKT2, Plasminogen Receptor with A C-Terminal Lysine 2; PRSS23, Serine Protease 23; RGS2, Regulator of G Protein-Signaling 2; RGS11, Regulator of G Protein-Signaling 11; RGS16, Regulator of G Protein-Signaling 16; RNASE1, Ribonuclease A Family Member 1; ROCK2, Rho-Associated Coiled-Coil Containing Protein Kinase 2; RPS26, Ribosomal Protein S26; S100A4, S100 Calcium-Binding Protein A4; SELP, Selectin P; SLC12A1, Solute Carrier Family 12 Member 1; SLC5A12, Solute Carrier Family 5 Member 12; SMAD3, SMAD Family Member 3; SMAD5, SMAD Family Member 5; SOX4, SRY-Box Transcription Factor 4; SPARC, Secreted Protein Acidic and Cysteine Rich; SPARCL1, SPARC Like 1; SPP1, Secreted Phosphoprotein 1; SYK, Spleen-Associated Tyrosine Kinase; TEK, TEK Receptor Tyrosine Kinase; THY, Thy-1 T-Cell Surface Antigen; TNF, Tumor Necrosis Factor; TGFBR1, Transforming Growth Factor Beta Receptor 1; TXNIP, Thioredoxin-Interacting Protein; UTRN, Utrophin; WFDC2, WAP Four-Disulfide Core Domain 2; WNT, Wingless-Type MMTV Integration Site Family; WT1, Wilms Tumor 1 Transcription Factor; YWHAH, Tyrosine 3-Monooxygenase/Tryptophan 5-Monooxygenase Activation Protein Eta; ZEB1, Zinc Finger E-Box-Binding Homeobox 1; ZEB2, Zinc Finger E-Box-Binding Homeobox 2

Jiang et al. [25] conducted the RNA-seq of six glomeruli microdissected from renal tissue of three living kidney donors and three IgAN patients. Glomeruli were separated by the remaining parenchyma to evaluate the specific gene expression. RNA-seq revealed 381 DEGs, of which 229 were upregulated and 152 were downregulated. The investigators identified genes related to aging, inflammation, and transcripts specific of the IgAN patients using the Database for Annotation, Visualization, and Integrated Discovery (DAVID) tool. Nine neutrophil extracellular traps transcripts (NETs) were specific to the IgAN. Three genes [Jun proto-oncogene (JUN), Fos proto-oncogene (FOS) and Plasminogen Activator, Urokinase (PLAU)], related to the neutrophil infiltration in glomeruli, overlapped with aging, inflammation, and IgAN. Two of them (JUN and FOS) increased the LL37 (or Cathelicidin AntiMicrobial Peptide, CAMP) gene involved in NETs (Table 2). The authors hypothesized that infiltration of neutrophils, inflammation factors, and aging genes induced the immune response and renal injury at the glomerular level.

Park et al. [26] conducted a gene expression study on microdissected glomeruli isolated from kidney biopsies of 14 IgAN patients with eGFR > 60 ml/min/1.73m^2^ and proteinuria less than 3 g/day, using the RNA-seq technique (Table 2). They found overexpressed genes involved in the inflammation [C–C motif chemokine ligand 3 (CCL3), C–C motif chemokine ligand 4 (CCL4), C-X-C motif chemokine ligand 16 (CXCL16)], B cell and Fcγ receptor (FcγR) pathways [Bruton tyrosine kinase (BTK), Mitogen-activated protein kinase 1 (MAPK1)], complement [Complement C4A (C4A), Complement C4B (C4B)], B4GALT1 (Beta-1,4-galactosyltransferase 1), and fibrosis [TGFβ, Tumor necrosis factor (TNF), SMAD family members (SMADs), Collagen type I alpha 1 chain (COL1A1)]. These findings showed that the first process of inflammation and fibrosis occurred in the glomeruli of IgAN patients. Pathway analysis evidenced canonical molecular pathways involved in B cell-signaling, chemokine signal transduction, and FcγR-mediated phagocytosis. Among the overexpressed genes, the authors focused on the role of Spleen tyrosine kinase (SYK), present in the glomerular transcriptome of IgAN patients and confirmed in the mesangium and infiltrating leukocytes by immunohistochemistry and immunofluorescence. Its presence increased in extracapillary lesions. The vital role of this phospho-SYK gene protein was demonstrated in vitro after stimulation of human mesangial cells with the serum of IgAN patients and even more after incubation of mesangial cells with IgA1 isolated from the serum of IgAN patients. In conclusion, SYK is one of the overexpressed molecules that are increased in mesangial cells during the kidney damage induced by the deposition of circulating Gd-IgA1 and immune complexes. Moreover, it contributes to the process of mesangial cell proliferation.

### Single-cell RNA-sequencing (scRNA-seq) technique in fresh whole renal tissue

Bulk tissue transcriptomic methods evaluate gene expression in a mixture of resident and non-resident cells in the kidney. In contrast, single-cell sequencing techniques evidence genomic patterns of single cells within the whole renal tissue. Next-generation sequencing or deep sequencing studies can be done using scRNA-seq or single nucleus RNA-seq methods. Another approach is to study the transcriptome profiling of the renal lesions using the surplus FFPE renal tissue obtained after the diagnostic course of the disease. The whole renal tissue, obtained from archival FFPE samples, is an underexploited source for molecular studies. Moving from the bulk RNA-seq of isolated glomeruli to the scRNA-seq makes it possible to analyze thousands of genes from large numbers of cell quantitatively. Bioinformatic tools have been developed to study the gene expression of specific cells in whole renal tissue. Still, the single-cell transcriptomic profile is more precise because it identifies a single-cell type gene expression. Thus, investigators can identify cell-specific gene expression involved in cell differentiation and the progression of the disease. In conclusion, scRNA-seq shows how the gene pattern of cells changes from the healthy status to the disease condition.

Zheng et al. [27] performed, for the first time, single-cell transcriptomics in kidneys and CD14^+^ peripheral blood mononuclear cells of 13 IgAN patients and six controls (Table 2). The gene expression patterns of the pathological mesangium, epithelium, and resident immune cells were evidenced after identifying nine distinct kidney cell types. High expression of genes characterized each cell cluster. The Joining Chain of Multimeric IgA and IgM (JCHAIN) gene was found to be upregulated in the mesangial cells that evidenced a potential correlation with the IgA deposition. Overexpression of genes encoding collagens, glycoproteins, and integrins was observed in the mesangium. Moreover, high levels of genes involved in the inflammation, such as cytokines and chemokines, were found. Finally, an increased cell-type-specific interaction gene expression between glomeruli and tubule-interstitium during the progression of the renal damage was observed. The interaction between mesangial cells and endothelial cells was based on the Angiopoietin 1 (ANGPT1)/ TEK Receptor Tyrosine Kinase (TEK) gene interaction. Moreover, the progression of the renal damage was expressed by macrophages and T-cell genes. Thus, this study generated a large volume of information, primarily prediction cell–cell interaction networks, not yet achieved in previous studies.

Tang et al. [28] conducted another RNA-seq study at the single-cell level. They identified DEGs in mesangial cells, endothelial cells, podocytes and tubular cells (Table 2). They found upregulated genes involved in cell proliferation, cell adhesion, and activation of inflammatory processes caused by deposition of Gd-IgA1 and immune complexes. Principally, upregulated genes [Metastasis-Associated Lung Adenocarcinoma Transcript 1 (MALAT1), Growth Arrest, and DNA Damage-Inducible Beta (GADD45B), SRY-Box Transcription Factor 4 (SOX4), and EGF-Like Repeats and Discoidin Domains 3 (EDIL3)] were involved in the mesangial cell proliferation and matrix accumulation. Then, upregulated genes involved in glomerular endothelial proliferation, cell–matrix adhesion, and leukocyte migration were observed. DEG-related signaling pathways were shown in tubular cells, of which proximal tubular cells were rich in TNF signaling, Interleukin-17 (IL-17) signaling, and leukocytes adhesion. These physiological and pathological processes are responsible for the progression of kidney damage. Moreover, intracellular signaling in mesangial cells and other cells demonstrated that the inflammatory process moving from the mesangial cell expanded to other glomerular cells. Finally, overt proteinuria induced a high expression of genes at the glomerular and tubular levels leading to fibrosis. The study showed upregulated genes responsible for immune cell infiltration (monocytes, macrophages, and dendritic cells) in the renal tissue of IgAN patients.

A large-scale scRNA-seq study was carried out by Chen et al. [29] in kidney biopsies from a small group of patients with varying glomerulonephrites, including IgAN (Table 2). Interestingly, a high expression of Lymphotoxin Beta (LTB) genes was found in IgAN and lupus nephritis. This gene may play a role in B cell activation to induce a high immune response. Furthermore, the increased expression of Tryptase Beta 2 (TPSB2) and Tryptase Alpha/Beta-1 (TPSAB1) contributed to more interstitial fibrosis. Finally, a high expression of CD74 within podocytes that indicated activation of Macrophage Migration Inhibitory Factor (MIF)/CD74 between podocytes and immune cells, was found. Moreover, upregulation of Chloride Intracellular Channel 1 (CLIC1) and Ribosomal Protein S26 (RPS26) was observed in mesangial cells associated with upregulation of JunB Proto-Oncogene (JUNB) in podocytes. Thus, after stimulation, the JUN gene was rapidly activated and dimerized with either JUN protein or Activator protein 1 (AP-1) to form the FOS protein. The gene JUNB, along with JUN and FOS, formed the upstream element of the TNF/TNF Receptor Superfamily Member 1 (TNFR1) pathway involved in inflammation, complement activation, coagulation cascade, and Renin–Angiotensin–Aldosterone System (RAAS) activation. Therefore, JUNB as reported by other investigators, may be a novel prognostic biomarker of IgAN. JUNB was upregulated in podocytes of IgAN patients. The primary limitation of this study was the low number of kidney biopsies included.

Although the data described in this section demonstrate the progress of technologies used in renal transcriptomics, there is an important limitation. Given the heterogeneity of methods used in a low number of IgAN patients, data must be taken with caution because, in many articles, results have not been validated in external cohort of patients with IgAN and other primary glomerulonephrites.

### Urinary biomarkers

Despite being widely used, available clinical biomarkers for IgAN have significant limitations, such as non-disease specificity, extensive variability, and lack of accuracy. In addition, these biomarkers are not related to molecular pathogenesis but rather represents a direct effect of organ damage. Thus, there is a need for new biomarkers that enable an earlier diagnosis, improved monitoring of the clinical course or response to treatment and targeted therapy [1].

Recently, MCP-1 and EGF were proposed as valuable biomarkers of IgAN progression and development of chronic histological lesions. Torres et al. [19] demonstrated that the predictive value of the EGF/MCP-1 ratio was significantly higher than that of EGF or MCP-1 alone, histological grade, creatinine clearance, or proteinuria. Recently, Ju et al. [18] demonstrated that the measurement of urinary EGF improved the ability to predict the renal outcome.

Lu et al. [30] reported that the serum- and glucocorticoid-inducible kinase (SGK1) expression was significantly upregulated in urine and renal tubules of IgAN patients with T1 and T2 renal lesions (Oxford histological classification). In contrast, its expression in serum did not increase significantly. Relationship analysis indicated that urinary and tissue SGK1 expressions were associated with heavy proteinuria and renal insufficiency in these patients.

Recently, Cox et al. [10], using the histological score of the Oxford classification integrated with transcriptomics of the total renal cortex, identified several genes able to characterize endocapillary-extracapillary proliferations [DEFA4, TNFAIP6, Fatty Acyl-CoA Reductase 2 (FAR2)] and chronic tubule-interstitial lesions (LTB, CXCL6, ITGAX) in IgAN patients. Two of them (TNFAIP6 protein for active renal lesions and CXCL6 protein for chronic lesions) were confirmed in the urine of an independent cohort of IgAN patients compared with non-IgAN patients and controls. These gene products could represent effective candidates for urinary biomarkers in clinical decision making.

Other urinary biomarkers for IgAN have been selected due to their significant correlation with major histological findings [31–33]. Among them, TGFβ [34], Neutrophil gelatinase-associated lipocalin (NGAL) [35], Kidney injury molecule-1 (KIM-1) [36, 37], Matrix metalloproteinase-7 (MMP7) [38], MCP-1 [39], EGF [40], and Uromodulin (UMOD) [41] have been largely described.

**Fig. 1:**
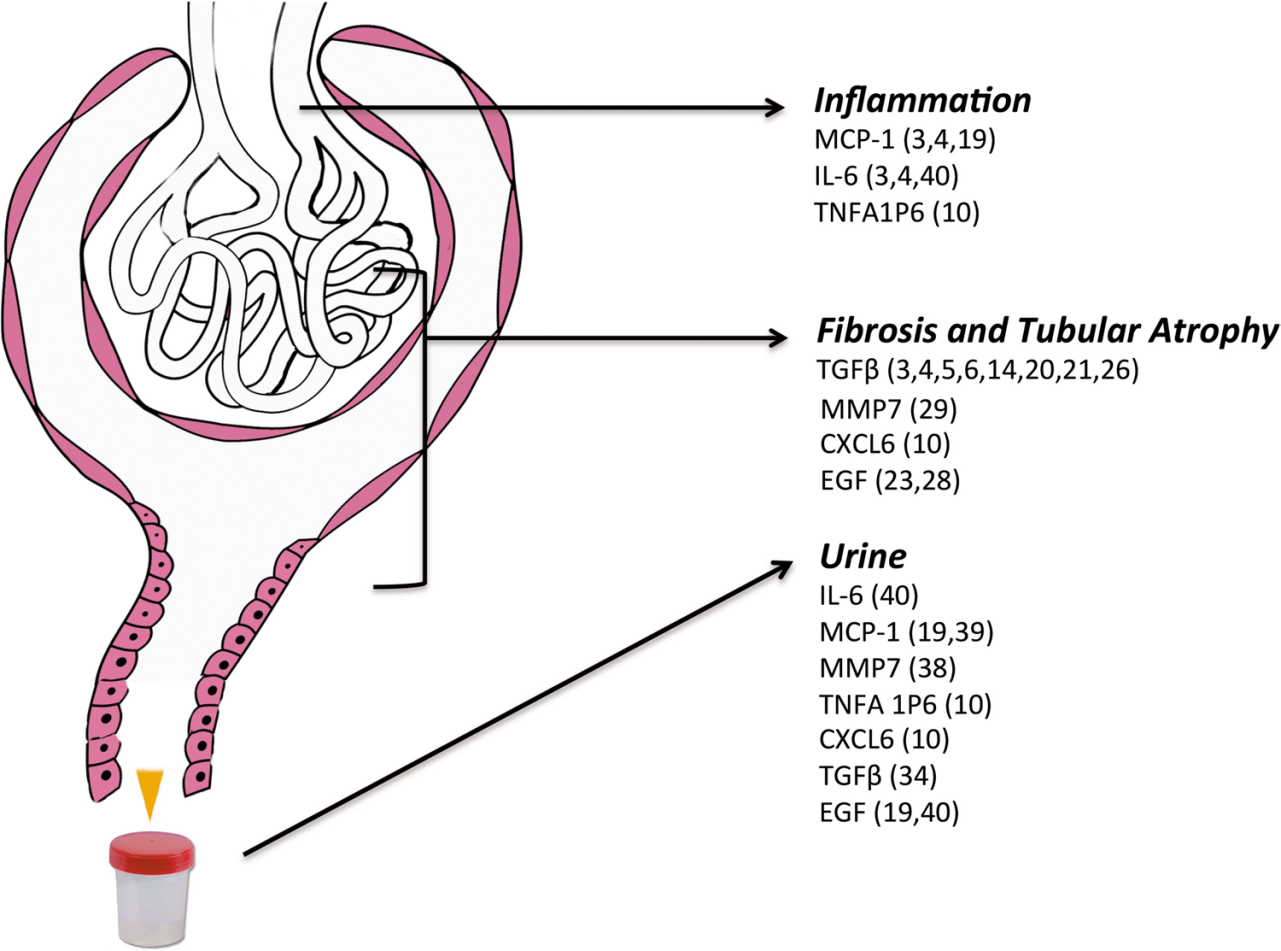
Schematic representation of integrative renal transcriptomic data and urinary findings observed in inflammation and fibrosis. Listed genes have been chosen on the basis of their recorded presence in kidney and urine studies. Numbers in parenthesis indicate references

Schematically, Fig. 1 illustrates upregulated genes involved in inflammation and fibrosis at glomerular and tubule-interstitial level, frequently observed in transcriptomic kidney studies and relative proteins detected in the urine of IgAN patients.

## Conclusions

The use of qRT-PCR, microarrays, and RNA-seq techniques in whole renal tissue and separated compartments of the nephron such as glomeruli and tubule-interstitium has clarified many aspects of renal damage in IgAN patients. Recently, the introduction of the scRNA-seq techniques has overcome the limitations of the previous methods because it is possible to study the whole renal tissue without the dissection of the nephron segments. It also better analyzes the cell-specific gene expression involved in cell differentiation. In conclusion, scRNA-seq can inform the investigator how the gene pattern of cells changes from the healthy status to the disease condition.

Transcriptome data have documented the participation of many specific genes in the mesangium when mesangial cells bind the deposited immune complexes. Moreover, other inflammatory molecules are produced by resident and non-resident cells that participate in the process of chronic inflammation and fibrosis. Some of these molecules may be the targets of old drugs, such as corticosteroids, renin–angiotensin–aldosterone blockers, and new drugs such as monoclonal antibodies. Finally, the transcriptome studies have uncovered some urinary biomarkers that can be used for the therapeutic monitoring of the IgAN patients during the follow-up, thus, avoiding a further kidney biopsy.

## Data Availability

Not applicable.
